# A spatially-distributed computational model to quantify behaviour of contrast agents in MR perfusion imaging

**DOI:** 10.1016/j.media.2014.07.002

**Published:** 2014-10

**Authors:** A.N. Cookson, J. Lee, C. Michler, R. Chabiniok, E. Hyde, D. Nordsletten, N.P. Smith

**Affiliations:** a Department of Biomedical Engineering, Division of Imaging Sciences & Biomedical Engineering, St. Thomas’ Hospital, King’s College London, London SE1 7EH, UK; b Department of Computer Science, University of Oxford, Oxford OX1 3QD, UK

**Keywords:** Magnetic resonance imaging, Myocardial perfusion, Contrast agent, Finite element method, Idealised modelling

## Abstract

Contrast agent enhanced magnetic resonance (MR) perfusion imaging provides an early, non-invasive indication of defects in the coronary circulation. However, the large variation of contrast agent properties, physiological state and imaging protocols means that optimisation of image acquisition is difficult to achieve. This situation motivates the development of a computational framework that, in turn, enables the efficient mapping of this parameter space to provide valuable information for optimisation of perfusion imaging in the clinical context. For this purpose a single-compartment porous medium model of capillary blood flow is developed which is coupled with a scalar transport model, to characterise the behaviour of both blood-pool and freely-diffusive contrast agents characterised by their ability to diffuse through the capillary wall into the extra-cellular space. A parameter space study is performed on the nondimensionalised equations using a 2D model for both healthy and diseased myocardium, examining the sensitivity of system behaviour to Peclet number, Damköhler number (*Da*), diffusivity ratio and fluid porosity. Assuming a linear MR signal response model, sample concentration time series data are calculated, and the sensitivity of clinically-relevant properties of these signals to the model parameters is quantified. Both upslope and peak values display significant non-monotonic behaviour with regard to the Damköhler number, with these properties showing a high degree of sensitivity in the parameter range relevant to contrast agents currently in use. However, the results suggest that signal upslope is the more robust and discerning metric for perfusion quantification, in particular for correlating with perfusion defect size. Finally, the results were examined in the context of nonlinear signal response, flow quantification via Fermi deconvolution and perfusion reserve index, which demonstrated that there is no single best set of contrast agent parameters, instead the contrast agents should be tailored to the specific imaging protocol and post-processing method to be used.

## Highlights

• A finite element model of myocardial MR perfusion imaging is proposed.
• A parameter space study is performed for models of both healthy and diseased tissue.
• Clinical metrics non-monotonic with respect to changes in extra-vascular diffusivity.
• Signal upslope a more robust clinical metric than peak signal value.

## Graphical abstract

**Figure:**
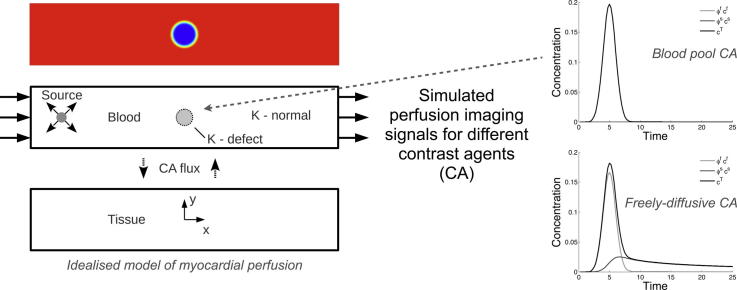

## 1 Introduction

There were more than 80,000 deaths due to coronary heart disease in the UK, during 2010 alone (Townsend et al., 2012). Associated treatment of the disease has been estimated at costing £1.8 billion, with an overall cost to the economy of £6.7 billion (Townsend et al., 2012). Earlier diagnosis of coronary heart disease therefore has the potential both to increase life expectancy and to reduce healthcare and other economic costs.

To achieve this, contrast agent (CA) enhanced magnetic resonance imaging (MRI) of myocardial perfusion (Coelho-Filho et al., 2013b) has been proposed as being able to provide a non-ionising, non-invasive, early indication of disease in the coronary circulation. This modality aims to yield a direct representation of the underlying physiological state of local perfusion (Barkhausen et al., 2004), by revealing areas of the myocardium that are receiving lower than normal blood flow. Common causes for these regional deficiencies in blood perfusion are stenosis of one or more of the main coronary arteries, and microvascular disease. Prolonged exposure of tissue to hypoperfusion can lead to functional consequences such as myocardial hibernation (Frangogiannis, 2003) and ultimately death of myocytes.

Various other imaging modalities exist for detecting these conditions, for example, Computed Tomography (CT) or MR angiography provide direct representation of any stenoses. However this structural diagnosis does not perfectly correlate with the functional diagnosis that perfusion imaging provides, nor does it detect microvascular disease. While nuclear medicine techniques such as Single Photon Emission Computed Tomography (SPECT) or Positron Emission Tomography (PET) can provide robust absolute quantification of perfusion, and even target specific metabolic consequences of tissue perfusion (Beller and Zaret, 2000), MR perfusion imaging realises significantly higher spatio-temporal resolution. This resolution enables the observation of localised perfusion defects which might otherwise be obscured. An additional advantage of using MRI is that the perfusion imaging can be combined with that of cardiac function, scar and other diagnostic targets within a single session, providing useful clinical flexibility. Finally, the lack of ionising radiation makes MRI safe and suitable for regular follow-up imaging of patients.

The basic protocol for performing cardiac perfusion imaging begins with the injection of a dose of contrast agent into a peripheral vein. This dye is then transported through the right ventricle, lungs and left ventricle, from where it is pumped into the myocardial circulation. Three imaging planes are taken along the short axis of the heart, at an approximate resolution of 1.2 × 1.2 × 10 (mm). The gadolinium-based CA boosts the MR signal of the blood relative to the surrounding tissue, such that areas which are under-perfused appear much darker in the image than those which are well-perfused. These images are then viewed by the clinician, who makes an expert judgement regarding the health of the patient’s heart based on the spatial distribution of contrast agent within the ventricle walls.

Beyond expert visual assessment is the potential for much greater information to be extracted from these images. There are now various methods that derive quantitative metrics of the perfusion state specifically and the disease state in general. For example, flow quantification is approached by calculating the maximum upslope of a the myocardial signal (Aquaro et al., 2013) or by using signal deconvolution techniques, either on segment-averaged data (Jerosch-Herold et al., 1998) or more recently on voxel-wise signals (Zarinabad et al., 2012). Other metrics include transmural perfusion gradients, which may discern between large-vessel and microvascular disease (Hautvast et al., 2011), and the perfusion reserve index which reflects the spare capacity in the myocardial circulation to adapt to stress conditions (Jerosch-Herold et al., 1998).

However, despite these promising aspects, the technique still possesses some limitations. Among the more significant drawbacks include: artifacts due to patient motion (Stegmann et al., 2005); quantification errors due to ECG trigger failures (Knesewitsch et al., 2013); a saturation of the signal response curve that prevents straightforward perfusion quantification (Ishida et al., 2011); reconstruction errors arising from under-sampled imaging sequences (Plein et al., 2007); dark rim artifacts (Di Bella et al., 2005); errors due to a combination of nonlinear signal response properties and partial volume effects in the sampled image. Furthermore, the diffusive nature of currently-used contrast agents such as Gadobutrol can confound quantification of blood flow and generates both false-positives and false-negatives in data interpretation (Barkhausen et al., 2004). These issues are compounded by the multi-modal causes of perfusion defects, from both arterial stenosis and diabetes-induced microvascular damage. Taken altogether, these considerations mean that experimental determination of optimal protocols is prohibitively expensive and time consuming.

To address these issues, existing models, typically lumped parameter in nature, have been used to investigate the transport of MRI contrast agents. These have been reviewed by Tofts et al. (1999) who used various ordinary differential equation (ODE) models and $T_{1}$-weighted MRI to estimate the kinetic parameters of diffusible tracers, in an attempt to standardise these models. However, ODE models of this transient transport process lack the ability to reproduce spatially-varying phenomena, such as the delineation of a perfusion defect boundary and effects due to the regional variation of cardiac physiology.

In previous publications, we have developed multi-compartment porous media models of myocardial perfusion (Michler et al., 2012, Cookson et al., 2012) and shown their suitability for simulating the phasic spatial/temporal changes in coronary perfusion. With these models, which couple 1D representations of the coronary arteries to 3D continuum porous models, both large-vessel and microvascular disease can be represented. The reduced area of an arterial stenosis can be specified in the 1D model and the permeability and porosity of the porous medium model varied locally to represent microvascular disease. However, to assess all of the necessary parameter variations of CA transport on a full multi-compartment, three-dimensional simulation would be extremely computationally expensive, producing large amounts of data that are difficult to interpret. Thus, in this study, a simplified approach to the problem is employed. It is important to clarify that the purpose of this model is to provide a simplified version of the 3D physiologically-realistic, multi-compartment porous medium model, so as to yield useful insights about its behaviour; as such it is not intended to be the simplest such reduction possible.

Applying this approach the principal aims of this study are: to provide a quantitative characterisation of the range of behaviour possible for different CAs, encompassing both current and possible, future compounds; to assess the likely errors associated with quantification of the perfusion state for different contrast agents parameters; and to understand the relationship between diagnostic precision and contrast agent properties with respect to the identification of a perfusion defect. The knowledge gained from these simulations will enable better interpretation of the results from the 3D simulations performed in physiologically-realistic geometries, and also to provide guidance for the use of different contrast agents.

## 2 Flow in a porous medium

### 2.1 Darcy flow

Porous media and poroelastic models have been previously used to simulate perfusion in the work of Huyghe et al., 1992, Vankan et al., 1997, May-Newman and McCulloch, 1998, Chapelle et al., 2010, Chapelle and Moireau, 2014, and more recently our studies applying this approach within physiologically-realistic left ventricle geometries (Michler et al., 2012, Cookson et al., 2012). In the latter papers a multi-compartment Darcy model is used, which contains multiple separate fluid networks, in order to permit a parameterisation that best captures the wide range of vessel length scales, and the widely-varying spatial distribution of blood flow that results. The work of Hyde et al., 2013a, Hyde et al., 2013b has demonstrated how this type of multi-compartment Darcy continuum model can be effectively parameterised from the discrete vascular data collected by van den Wijngaard et al. (2013).

However, given that the principal transfer of contrast agent to the tissue occurs in the capillaries, it is sufficient for the purposes of this study to consider here a standard, single-compartment porous medium. The porous medium, in the domain *Ω*, therefore contains two phases, fluid and solid (superscripts *f* and *s*, respectively), which have volume fractions, or porosities *ϕ*, defined as follows:

(1a) $$\phi^{f}=\frac{V^{f}}{V^{\Omega }}$$

(1b) $$\phi^{s}=\frac{1-V^{f}}{V^{\Omega }}$$

where

(2) $$V^{R}=\int_{R}\;\mathrm{dR}$$

for a given region *R*. Assuming saturation, these definitions imply that $\phi^{f}+\phi^{s}=1$.

The flow of blood through this medium can be described by Darcy’s law:

(3a) μw+K·∇p=0inΩ,

(3b) ∇·w=φinΩ,

where w and *p* denote the Darcy (or perfusion) velocity and pore pressure, respectively. The remaining quantities are μ>0, the dynamic viscosity of the fluid, K, the permeability tensor (symmetric positive definite) of the porous solid, and *φ*, the volumetric source term. Note that the Darcy velocity is related to the fluid velocity $u^{f}$ in the following way:

(4) $$w=\phi^{f}u^{f}$$

which indicates the interchangeability of velocity and porosity for a given Darcy flow. Some details of the numerical method used to solve this system are given in Section 4.1.

### 2.2 Transport of passive scalar in a porous medium

Broadly there are two types of contrast agents in which we are interested. The first are blood-pool CAs (intravascular CAs), which bind to blood proteins, typically albumin, and are therefore unable to penetrate the vessel wall and hence are confined to remain within the vessel lumen. The second are freely-diffusive CAs (extracellular CAs) which are able to diffuse through the vessel walls into the extracellular space of the myocardial tissue.

#### 2.2.1 Governing equations

Using the velocity field obtained from Eq. (3a), (3b), the transport of contrast agent by the blood can be determined. In the general case, two equations are required. The first is an advection–diffusion equation to describe the transport of CA in the blood, and the second is a diffusion equation that describes the movement of CA within the extracellular space.

In the fluid phase the transport of CA is described by the following advection–diffusion equation (Beaudoin et al., 2007):

(5a) $$\frac{\partial \phi^{f}c^{f}}{\partial t}+\nabla \cdot (\phi^{f}u^{f}c^{f})=\nabla \cdot (\phi^{f}D^{f}\nabla c^{f})-\overset{¯}{f}+q$$

The mass concentration of CA, defined relative to the phase volume, is denoted *c*. The product ϕc therefore gives the value of concentration per total volume. Conversely, the source term *q* is defined per unit total volume, as the amount of CA injected into the patient is independent of the myocardial porosity. The velocity $u^{f}$ is determined by solving Eq. (3a), (3b) and $D^{f}$ is the diffusion coefficient of CA in the blood. The additional sink term $\overset{¯}{f}$ accounts for the transfer of CA from the blood into the tissue. Once in the tissue, the transport of the CA through the extracellular space is described by the following diffusion equation:

(5b) $$\frac{\partial \phi^{s}c^{s}}{\partial t}=\nabla \cdot (\phi^{s}D^{s}\nabla c^{s})+\overset{¯}{f}$$

where $\overset{¯}{f}$ now appears as a source term and $D^{s}$ is the diffusion coefficient of CA in the tissue.

#### 2.2.2 Through-wall flux of contrast agent

There are several possible interface conditions that could be chosen for the transport of CA across the vessel wall, the simplest being continuity of concentration. The form taken here assumes that the wall can sustain a concentration gradient, and that the flux through it occurs at a rate proportional to that gradient across the interface. At the microscale the flux, *f*, is therefore:

(6) $$f\propto (c^{f}-c^{s})$$

At the macroscale, which is a continuum representation of the average behaviour of many vessels within a given volume element, the same proportionality holds. Following Gerke and van Genuchten (1993), the mass transfer term becomes:

(7) $$\overset{¯}{f}=\alpha \phi^{f}\phi^{s}(c^{f}-c^{s})$$

The constants of proportionality are thus $\alpha \text{,}\;\phi^{f}$ and $\phi^{s}$. In an analogous way to the other concentration gradient term $\nabla D\phi^{f}\cdot \nabla c$ in Eq. (5a), porosity does not directly multiply the concentration values. This is because the volume of a pore can be increased while keeping surface area constant (through an appropriate reconfiguration of the pore geometry), which preserves the value of the flux. In addition, this relationship naturally enforces the observation that should the porosity of either phase go to zero, then no mass transfer to/from that phase can occur. Finally, other properties being equal, the case of $\phi^{f}=\phi^{s}=0.5$ yields maximal flux between the two phases.

The parameter *α* represents both the permeability of the vessel wall to the contrast agent and the efficiency of the vascular geometry at mass transfer. The value of this constant will in future be determined from experimental data. A value of α=0 corresponds to a blood-pool CA, and α>0 the freely-diffusive agents. Therefore, the qualitative binary distinction between these two classes of CA is condensed into a single parameter value in the model. Finally, it is assumed that the vessel wall membrane is equally permeable in both directions of mass flux.

### 2.3 Nondimensionalisation

A nondimensionalisation of the governing equations is performed to provide insight into the key physical processes of the system, as well as to reveal the main relationships between the various parameters. Furthermore, by reducing the set of parameters to the minimum necessary to describe the system, the parameter space study is faster to perform and the results more straight forward to analyse.

Before performing the nondimensionalisation, some additional assumptions are made regarding the material fields. In this study, both spatial and temporal gradients of porosity and the diffusion coefficient, *D*, are ignored. Future studies will allow these fields to vary so as to enable more specific parameterisations of different disease states. In the Darcy model, with unit inflow/outflow boundary conditions, the velocity field is set to be divergence free. Under these conditions the equations simplify to

(8a) $$\frac{\partial c^{f}}{\partial t}+u^{f}\cdot \nabla c^{f}=D^{f}\nabla^{2}c^{f}-\alpha \phi^{s}(c^{f}-c^{s})+\frac{q}{\phi^{f}}$$

for the blood, and

(8b) $$\frac{\partial c^{s}}{\partial t}=D^{s}\nabla^{2}c^{s}+\alpha \phi^{f}(c^{f}-c^{s})$$

for the extracellular space. The following scalings are used to nondimensionalise the system (following the example of Shipley and Chapman (2010)), $u=\mathrm{Uu}\prime \text{,}\;t=\frac{d}{U}t\prime \text{,}\;c=\mathrm{Cc}\prime$ and x=dx′, where *U* is a typical velocity, *C* a typical concentration and *d* an inter-capillary length. This produces:

(9a) $$\frac{\partial c^{f}}{\partial t}+u^{f}\cdot \nabla c^{f}=\frac{1}{\mathrm{Pe}}\nabla^{2}c^{f}-\mathrm{Da}(1-\phi^{f})(c^{f}-c^{s})+\frac{q}{\phi^{f}}$$

(9b) $$\frac{\partial c^{s}}{\partial t}=\frac{D_{r}}{\mathrm{Pe}}\nabla^{2}c^{s}+\mathrm{Da}\phi^{f}(c^{f}-c^{s})$$

where all variables are now the nondimensional versions but with the prime omitted for conciseness. A summary of the four dimensionless parameters is given below.

1. Peclet number $(\mathrm{Pe})=\frac{\mathrm{Ud}}{D^{f}}$, characterises the relative time scales of advective and diffusive processes in the blood.
2. Damköhler number $(\mathrm{Da})=\frac{\alpha d}{U}$, characterises the relative rates with which contrast agent passes through the vessel wall and is advected past it by the blood.
3. Diffusivity ratio ($D_{r})=\frac{D^{s}}{D^{f}}$, indicates the relative importance of diffusive processes in the intra- and extravascular spaces.
4. Fluid porosity $\phi^{f}$, the volume fraction of the porous medium occupied by the blood.

A summary of the numerical methods used to solve this coupled system is given in Section 4.2.

## 3 Model setup and goals

### 3.1 Tissue model

**Fig. 1:**
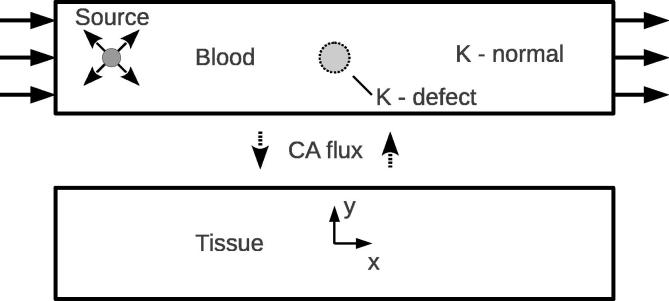
Schematic showing the 2D idealised system. Inlet and outlet flow boundary conditions are applied, with a point source applied in the transport system. The size and location of the small defect is marked in the blood domain.

Fig. 1 shows the layout of the 2D domain, marked with the location of the point source and direction of flow. Two different configurations will be used to account for healthy and diseased cases. The healthy myocardium is represented by a spatially constant isotropic value of permeability, K=I. For the diseased case, the associated regional perfusion defect is characterised by very low flow within a localised area of the myocardium. This reduction in flow rate is accomplished by imposing a circular region of near-zero permeability in the Darcy model, as implemented in Nolte et al. (2013). For reasons of numerical compliance, K cannot be set exactly to zero. Additionally a transition region is defined that spans two or three elements, which is accomplished using a hyperbolic tangent function. The precise form of this masking function is given by Eq. (10), the parameters of which can be tuned to alter the size and sharpness of the boundary region of the defect:

(10) $$K_{\mathrm{mask}}=0.5(\tanh(-50{\left(x^{2}+y^{2}\right)}^{1/2}+15)+1)$$

This is applied to the unit permeability field in the following way:

(11) $$K=I\;(1-\gamma K_{\mathrm{mask}})$$

**Fig. 2:**
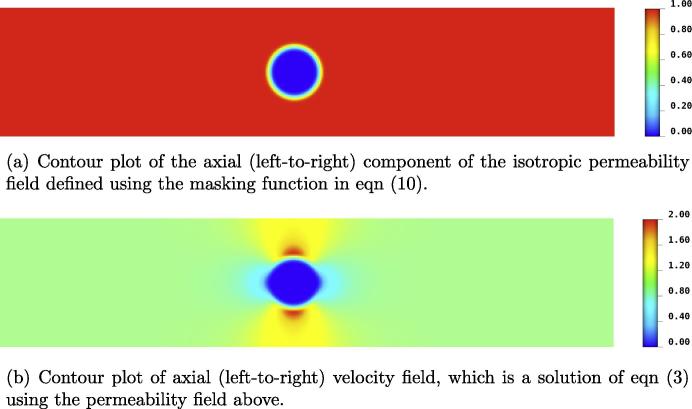
The perfusion defect is modelled by a circular region of near-zero K. Due to the fact that K is not exactly zero in this region, a very small flux passes through the defect region. This means that the path of least resistance to the flow is a compromise between distance travelled and resistance offered by the permeability field, and hence means that the circular boundary of the defect is not perfectly reproduced in the velocity field.

In this study, γ=0.999 and the resultant permeability and axial velocity fields are shown in Fig. 2. Three sizes of defect are investigated with radii 0.065,0.17 and 0.29, within a domain one unit in width and five units in length.

### 3.2 Aims

The freely-diffusive nature of some contrast agents means that a quantity of CA becomes stored in the tissue for time scales longer than the injection and clearance of the CA in the blood pool, confounding the correct interpretation of the underlying physiological state. In clinical terms, during perfusion imaging, a false-negative result would be that hypoperfusion was present, yet there were no identifiable perfusion defects in the MR images. A false-positive is the opposite to this, whereby blood perfusion of a healthy subject is under-estimated. Both of these scenarios have the potential to occur as a consequence of the temporary storage of CA in the tissue.

For the case of a false-positive, there are two contributing causes. The first is a local enhancement of the signal at the point where CA diffuses into the tissue, producing an over-estimation of perfusion. The second cause is the removal of CA from the fluid into the tissue leaving less CA available downstream and therefore, for equal blood volumes, the downstream portion will emit a lower signal, producing a false-positive effect of under-estimation. These two effects can combine to produce a disparity in signal between two regions of tissue that are identically well-perfused, complicating quantification of blood flow.

The under-estimation due to this effect will clearly depend on the Damköhler number, and the downstream distance of the sampling point. For small values of *Da*, this effect is likely negligible, but larger values have the potential to create significant errors in the quantitative interpretation of the perfusion state.

The false-negative effect occurs when a freely-diffusing contrast agent diffuses through the tissue phase from a region of healthy perfusion to a region of under perfusion, thereby producing signal in an unperfused area which should have none. The previously-described models for healthy and diseased tissue simplify the analysis of results by separating these two effects.

### 3.3 Input bolus specification

In the clinic, the contrast agent is generally injected into a peripheral vein as quickly as is practicable, so as to approximate a Dirac delta function, though in reality it will be a triangular or top-hat function. Alternative injection strategies have been proposed, for example the injection of a pre-bolus that is a fraction of the main bolus in order to enable signal calibration (Ishida et al., 2011), however an analysis of this approach is outside the scope of this work.

After having travelled to, and through, the right ventricle and lungs, this input bolus becomes smeared by dispersion. For an MRI region-of-interest located in the blood pool of the left ventricle, the concentration time-signal of this bolus now approximates a slightly-skewed Gaussian function. This signal is known as the arterial input function, as it is taken to be the input seen by the myocardial circulation, and it can be used in deconvolution techniques for quantifying perfusion (Hautvast et al., 2012). As a first approximation to this injection and subsequent dispersion, and neglecting the small skewness of arterial input function, a transient Gaussian source term, Eq. (12), is used in the advection–diffusion equation.

(12) $$q=\frac{1}{\sigma \sqrt{2\pi }}e^{-\frac{1}{2}{\left(\frac{t-T_{\mathrm{peak}}}{\sigma }\right)}^{2}}$$

This source term is applied at a single mesh node (0.0, −2.0) i.e. on the centreline 0.5 units from inflow boundary. The variance of the bolus is $\sigma^{2}$, which reflects the speed of the injection into the system, *t* is time and $T_{\mathrm{peak}}$ is the mean of the Gaussian which sets the time value at which the peak value of the bolus occurs. For ease of analysis the integral value of the source term is kept at 1.

### 3.4 Signal response

The equations outlined in Section 2.2 calculate the evolution of contrast agent concentration in fluid and solid phases. One confounding factor to absolute quantification of perfusion is the fact that for a given imaging voxel, the resultant signal intensity is determined by an unknown mixture of signals from the fluid and solid. Therefore, we wish to calculate the total signal produced for these values of concentration. Typically the contrast agent signal response curves are formed by a linear region, followed by an almost-complete saturation, in which further increases in concentration yield only very small increases in signal intensity.

In this study it is mainly assumed that the imaging is performed within the linear range of the signal response curve. This enables the greatest applicability of the results and reduces the amount of confounding information in their analysis. Furthermore, a full parameterisation of the nonlinearity would be, in combination with the existing PDE parameter space, too large to consider here. However, in order to provide some insight as to the likely errors that can arise when assuming linearity, in Section 8.1 a set of three nonlinear response curves, taken from (Hsu et al., 2008), are applied to a selection of the results. See also the work of Ishida et al. (2009) for further indicative measurements of nonlinear signal responses.

The total porosity-weighted concentration, $c^{T}$, is defined in Eq. (13),

(13) $$c^{T}=\phi^{f}c^{f}+\phi^{s}c^{s}$$

and which can be taken to represent directly the MR signal intensity for a linear response curve, without any further post-processing.

Even for this simplified model, a key question is how to best draw conclusions from the wealth of spatially and temporally-varying data that these simulations produce. Therefore the following clinically-motivated metrics are used to reduce this information to a manageable form for analysis.

A single sample point, located at the centre of the domain, is specified and the three concentration values of porosity-weighted fluid concentration, porosity-weighted tissue concentration extracted and porosity-weighted total concentration. From the resultant time series, the upslope and peak value of concentration are calculated. The upslope is defined as the maximum gradient of the curve between t=0 and the time at which the peak value occurs (Aquaro et al., 2013).

The value of concentration in the defect displays a large degree of spatial inhomogeneity, therefore sampling at a single point is less useful in the diseased simulation in comparison to the healthy case. For the diseased simulations the average value of concentration, Eq. (14), is calculated for this region, defined using the same masking function as for the permeability field.

(14) $${\overset{¯}{c}}_{\mathrm{defect}}=\frac{\int_{\Omega }K_{\mathrm{mask}}(\phi^{f}c^{f}+\phi^{s}c^{s})\;d\Omega }{\int_{\Omega }K_{\mathrm{mask}}\;d\Omega }$$

Errors associated with the mask boundary spanning two elements are assumed to be unimportant. As with the healthy case, similar properties of the time-varying average signal are extracted.

## 4 Numerical methods

The finite element method is used to solve all the models presented in this paper, all of which are implemented within an in-house parallelised, multi-physics code, CHeart (Michler et al., 2012, McCormick et al., 2013, Nordsletten et al., 2010). A brief description for each model component follows.

### 4.1 Darcy perfusion model

The 2D spatial domain is discretised using linear Lagrange quadrilateral elements, of size 0.02 units, with velocity and pressure represented by quadratic Lagrange basis functions. This system is solved using the primal, or pressure, formulation, which is obtained by the substitution of the Darcy flux equation into the continuity equation. No-flux boundary conditions are imposed on the long edges of the domain, with fixed unit inflow/outflow velocity conditions applied to the short edges, for both the healthy and diseased cases. For full numerical details see (Michler et al., 2012).

### 4.2 Coupled advection–diffusion-reaction transport model

Similar to the Darcy solution, the spatial domain is discretised using linear Lagrange quadrilateral elements, as are the concentration and source variables. The input velocity field u is represented on the same quadratic basis on which it was solved. To ameliorate the numerical instability of the associated advection operator, the Streamline-Upwinding Petrov–Galerkin (SUPG) stabilisation scheme (Brooks and Hughes, 1982) is used. The discrete forms of these equations are assembled into a single matrix system, therefore solving for $c^{f}$ and $c^{s}$ simultaneously. The time integration of this global system from a zero initial condition is performed implicitly, using a backward Euler scheme. All four boundaries are set to a zero Neumann value, which enforces a condition of zero diffusive flux.

## 5 Results

### 5.1 Parameter space

It is the aim of this study to understand the behaviour of this system for a wide range of parameter values, which is a superset of values that includes those for contrast agents currently in use in clinical practice and experiments. This choice of range will provide a reference for any future contrast agents that might be developed. However, there are still limits to these ranges beyond which it is unlikely to be necessary to simulate. The particular choices of parameter values are justified below.

#### 5.1.1 Peclet number

The value of *Pe* is likely to be large for these contrast agents, however to encompass the conditions experienced during a full cycle of pulsatile flow, during which velocities will at times be very low, the following range of values is used 10–10,000.

#### 5.1.2 Damköhler number

A Damköhler number of 0 corresponds to the blood pool contrast agent. Results will be presented for *Da* in the range 0–100.

#### 5.1.3 Diffusivity ratio

It is unlikely that the diffusion coefficient of the CA in the tissue will be many orders of magnitude higher than in the blood. Therefore, the range of diffusivity ratio under investigation will be 0.01–10.

#### 5.1.4 Fluid porosity

A small range of porosity values are examined here, 0.14–0.18, which encompasses the capillaries, but also assesses the impact of increased porosity due to the presence of larger blood vessels, if a contrast agent were developed that permeated through the walls of these vessels.

## 6 Healthy case

**Fig. 3:**
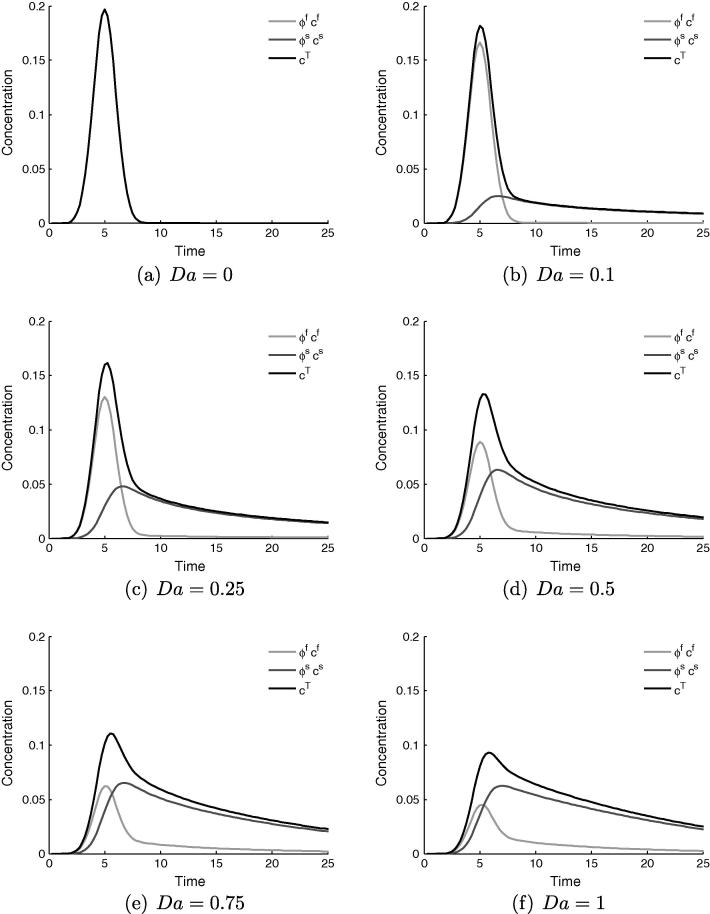
Time variation (seconds) of concentration measured at the central sampling point in the healthy tissue model, showing the relative contributions of the fluid and solid phases to the total observed concentration. For all cases Pe=1000,Dr=1 and $\phi^{f}=0.14$, with *Da* varying.

Fig. 3 shows a representative set of time series data for the concentration value at the sampling point, for varying values of *Da* in the range 0 to 1. The total concentration is displayed in black, with the contributions from the fluid and tissue in light and dark grey respectively.

Fig. 3a, where Da=0, corresponds to a blood-pool contrast agent, which manifests as zero tissue signal and coincident concentrations for the fluid and total. The effect of increasing diffusivity into the extracellular space to Da=0.1, Fig. 3b, is small, at which value there a small amount of contrast agent in the tissue. This storage lengthens the tail of the previously-Gaussian concentration distribution, and introduces a kink at which the downward gradient changes sharply. This kink marks the abrupt change in composition of the total concentration value that occurs when the bolus in the fluid has passed by the sampling point, leaving only a contribution from the tissue. This effect becomes more pronounced as *Da* is increased up to a value of one, in Fig. 3f. It is clear from these figures that a large change in system behaviour, both qualitative and quantitative, occurs between the values of Da=0.1 and Da=1.0.

### 6.1 Estimation of parameters for current contrast agents

**Fig. 4:**
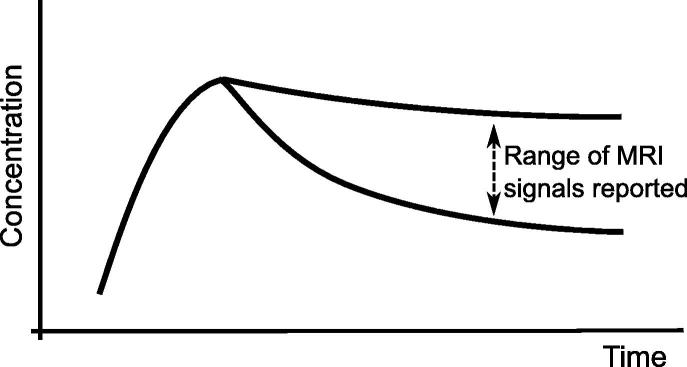
An idealised schematic representation of the range of MRI perfusion signals reported in the clinical literature. Comparison with simulation results suggests *Da* is in the range 0.25–2.0.

A survey of medical literature (Kelle et al., 2010, Chiribiri et al., 2011, Makowski et al., 2010, Köstler et al., 2008, Ritter et al., 2006, Su et al., 2007, Jerosch-Herold et al., 2004) was performed to identify the typical pointwise signal intensity curves measured during contrast agent enhanced MR perfusion imaging. These curves have been amalgamated into a single idealised representation in Fig. 4 to illustrate the possible range of behaviour. Given that only *Da* significantly affects the tail of the signal, the other parameters tending to have more symmetrical effects, user observation of the signal can be used to estimate *Da*, and be done so independently of the other parameters. Based on this comparison, *Da* is estimated to be in the range 0.25–2.0.

With regard to the diffusivity ratio, it is assumed that the diffusion coefficient of the CA in blood is roughly the same as in the extracellular fluid. However, the effective, or observed, diffusion coefficients at the macroscale in the porous medium will take different values. Just as for the Damköhler number, the effective diffusion coefficient of the CA is governed by more than just its chemical properties, with the geometric and physical properties of the vessels and tissue also influencing the macroscale behaviour of the CA. In particular, the pore geometry and porosity of the porous phase will determine the ease with which a molecule can diffuse within the bulk volume (Grathwohl, 1998). Other things being equal, the ratio of porosities in the two pore spaces will determine the ratio of their diffusivities in the bulk volume. A range of 15–33% porosity of the extracellular space has been used in bi-domain models of electrical activation of cardiac tissue (Stinstra et al., 2006), which therefore implies a porosity of the total volume in the range 12–28%. This suggests a *Dr* of 1–2, however, allowing a factor two of uncertainty to account for the two different pore morphologies, suggests a plausible range of values for *Dr* is ≈0.5–5.0.

It should be noted that the model presented here is somewhat phenomenological in terms of the parameters *Da* and *Dr*. Any geometric and other complexities are absorbed into the parameter values, to be more precisely determined from controlled experiments and formal parameter estimation techniques, rather than using a mechanistic approach to derive estimates based on idealisations of the pore or cell structure. The sensitivity of the system behaviour to the various parameters will be examined in the following sections, with reference to plots of the key signal characteristics of peak value and signal upslope.

### 6.2 Effect of varying *Da* and $\phi^{f}$

**Fig. 5:**
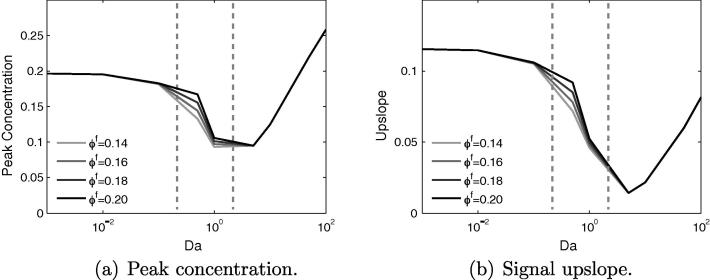
Variation of concentration signal properties with *Da* and $\phi^{f}$ for the healthy tissue model, with Dr=1. Both peak value and upslope are largely insensitive to changes in the fluid porosity, for all values of *Da*. Estimated current values of *Da* indicated by dashed vertical lines.

In Fig. 5a and b, showing the change in peak concentration and upslope with *Da* and $\phi^{f}$, it is clear that in the range of porosity examined, these signal characteristics are largely insensitive to the specific value of $\phi^{f}$. Therefore, in subsequent plots, $\phi^{f}$ will be fixed to a value of 0.14 and only the remaining three parameters varied.

More interesting is the non-monotonic behaviour around Da≈7.5, which occurs for the following reasons. As *Da* is increased from a value of 0 up to 7.5, a “steal effect” becomes increasingly significant as an increasing amount of CA is taken into the tissue upstream of the sample point. This reduces the amount of CA that is transported downstream by the flow to the sample point, thereby reducing the peak value. This effect causes the potential for false-positive readings to occur.

**Fig. 6:**
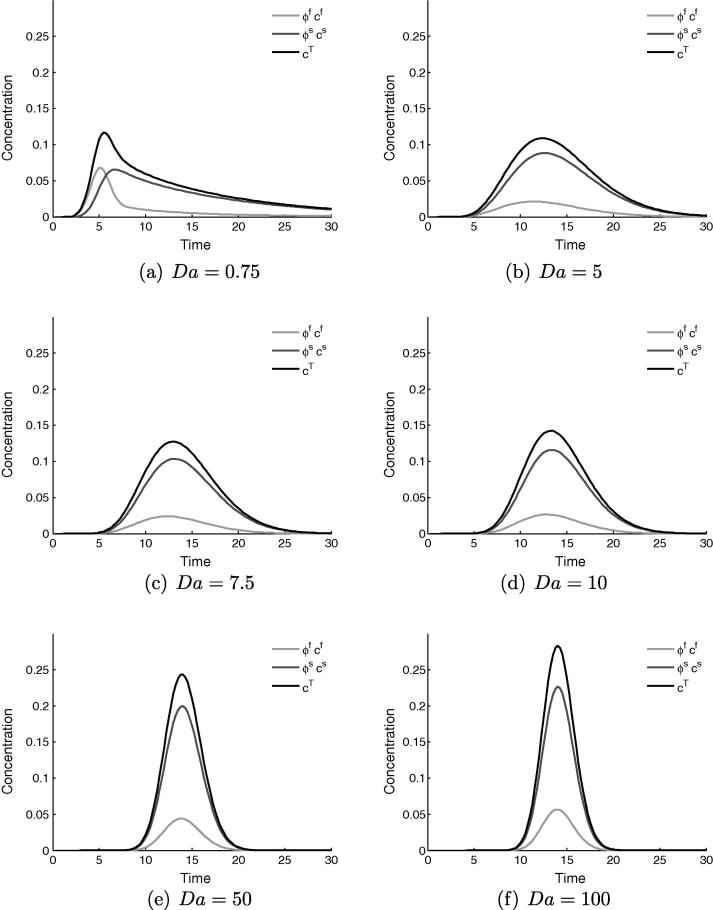
The profile of the concentration signal changes drastically in the range of Da=1–7.5. From the Gaussian curve with long tail at Da=0.75, increasing the value of *Da* gradually causes a shift back to a symmetric Gaussian distribution, except that the principal transport downstream now occurs in the tissue phase. This shift in signal shape permits an estimation of the values of *Da* encountered in clinical imaging.

As *Da* is increased beyond a value of 7.5, the peak concentration rises again. In these cases, the upstream steal effect is so strong that a large portion of the contrast agent is taken from the fluid into the tissue, where it is stored before slowly leaking back into the vessels. The majority of the signal measured downstream therefore derives from the tissue phase, the contrast agent having diffused through the tissue to the sample point. This is confirmed by the composition of the total signal in Fig. 6.

The non-unique nature of peak concentration and upslope means that for inverse approaches, these properties by themselves are unsuitable for identifying the value of *Da*. However, comparison of the signals for two values of *Da* either side of Da=7.5 shows that the shape of the curve varies greatly, in particular in the tail.

For Da<7.5 the curve is approximately Gaussian in the front, with a skewed Gaussian profile in the tail, the length of which is dependent on *Da* and *Pe*. This profile occurs because the concentration is transported by the flow, from an initial Gaussian distribution, which is shifted, scaled and stretched due to both the diffusion in the blood, and diffusion into the extracellular space. However, for Da>7.5 the signal is more symmetric in shape, but also stretched over a longer time period, as the concentration is moving into the observation zone principally through diffusion.

The upslope of the concentration signal also reduces with increasing *Da* in the range 0–1. This is again due to the upstream steal effect. The gradient of the signal reduces because the quantity of contrast agent that diffuses into the extracellular space is also proportional to the concentration difference, and not only to *Da*. Therefore, the peak of the input bolus is reduced to a greater extent than the rest of the curve. As *Da* increases, this reduction in the gradient is accentuated.

Finally, within the range of 0.25<Da<2, it is observed that the greatest sensitivity to changes in $\phi^{f}$ occurs for Da≈0.7, yielding up to 25% change in signal properties. Therefore from the perspective of reducing this sensitivity to changes in $\phi^{f}$, as will occur due to regional, physiological variations and during myocardial contraction, the ideal value of *Da* lies either below or above this value, where any changes in signal characteristics become negligible.

### 6.3 Effect of varying *Da* and *Dr*

**Fig. 7:**
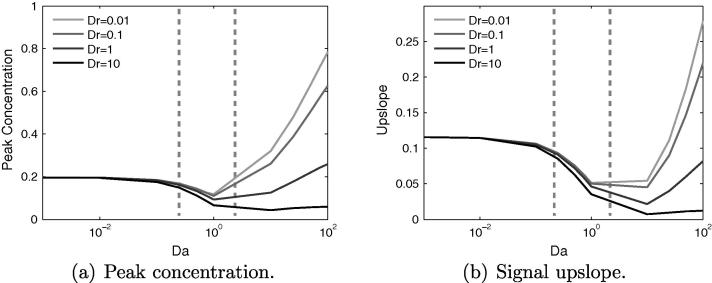
Variation of concentration signal properties with *Da* and *Dr*. For Da<1 these properties are insensitive to changes in *Dr*. However, for Da>1, they become increasingly sensitive with further increases in *Da*. Estimated current values of *Da* indicated by dashed vertical lines.

Fig. 7 shows the variation of peak concentration and upslope with varying *Da* and *Dr*. For Da<0.1, the curves are virtually indistinguishable, but a small difference emerges as *Da* is increased by an order of magnitude. Beyond Da=1 the curves rapidly diverge, and for Da=100 there is an order of magnitude difference in peak concentration between Dr=10 and Dr=0.01, and the situation is similar for the upslope.

This greater sensitivity to *Dr* for values of Da>1 is due to the much greater quantity of CA that enters the tissue for these parameter values. Altering *Dr*, and by implication $D^{s}$, will then have a more noticeable impact on the total signal. Below Da=0.1, there is simply too small a contribution from the $c^{s}$ for any changes in it to be significant to the total signal.

For Da=1, increasing *Dr* from 0.01 to 1 yields less than a 10% change in the upslope, though the peak value reduces by up to 20%. These changes in signal characteristics have the virtue of being bounded with regard to the errors they might propagate. Conversely, increasing *Dr* above a value of one, could have a large and potentially unbounded impact on the signal characteristics. An order of magnitude increase in *Dr* from 1 to 10, brings about a 22% reduction in the upslope and a 29% reduction in peak concentration, which is due to the CA diffusing more rapidly away from the sampling point. Increasing *Dr* further will only amplify this effect.

The curves once again display the non-monotonic behaviour seen in Fig. 5 for all values of *Dr*, though the effect is muted when Dr=10. This is because the contrast agent rapidly enters the extracellular space, where it then undergoes rapid diffusion, counteracting the coalescing effect of storage in the tissue that otherwise occurs for small *Dr*. In essence, the larger the diffusion coefficient in the tissue, the closer this scenario of CA transport in the tissue will mimic that transport in the blood. The key cause of the non-monotonic behaviour is the much slower transport away from the point source that occurs in the tissue.

In the parameter range, 0.25<Da<2 and 0.5<Dr<5, estimated to be relevant to current contrast agents, both signal properties are far more sensitive to changes in *Da* than to *Dr*. Specifically, there is a doubling of peak value between the extremes of *Da* but only a maximum 25% difference possible when altering *Dr* alone. Similarly for signal upslope, there is a factor 3.5 change between the limits of *Da* and again a 25% difference possible when varying *Dr* alone.

### 6.4 Effect of varying *Da* and *Pe*

**Fig. 8:**
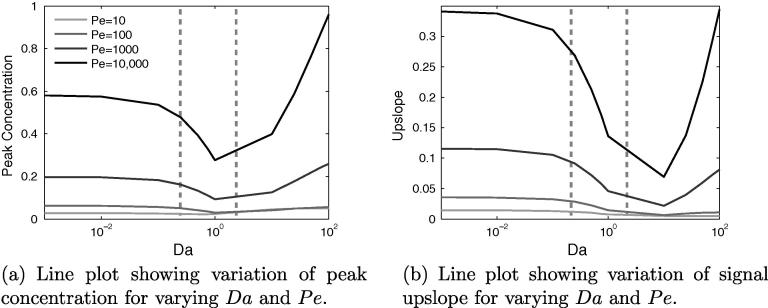
For the healthy model, much of the interesting variation in signal properties occurs with changes to *Da* and *Pe*. Estimated current values of *Da* indicated by dashed vertical lines.

Fig. 8a and b shows that both of the signal properties change in broadly the same way when varying *Da* and *Pe*. A higher value of *Pe*, for a given value of *Da*, produces both a higher peak value and a higher upslope. Upslope and peak value are strongly correlated, however, though a larger peak value will generally require a larger gradient, their minimum points for these properties occur at different values of *Da* – for peak concentration at Da=1, whereas for upslope Da≈10.

For the whole range of *Da*, the curves for each *Pe* remain distinct, in contrast to $\phi^{f}$ and *Dr*. This is because the influence of Peclet number is in the fluid phase, and hence its impact is not dependent on there being a non-negligible quantity of contrast agent in the tissue phase, as is the case for *Dr*. A lower value of *Pe* causes greater lateral diffusion of the CA bolus during transit to the sample point, consequently reducing the quantity of CA that reaches this point.

In quantitative terms, both properties roughly triple with each order-of-magnitude increase of *Pe*. For a given *Pe*, they are most sensitive to *Da* in the range 0.1–1.0. For Da<0.01 the properties are highly insensitive to changes in *Da*, as very little CA is diffusing into the tissue, and so it is a case of small changes to a small quantity. Within the range 0.25<Da<2 peak concentration begins to display non-monotonic behaviour, whereas signal upslope does not, suggesting that for current contrast agents, quantification methods based on upslope could be more reliable.

### 6.5 Summary

The contrast agents currently used are estimated to exist in the trough of the graphs, where there is a non-monotonic change in signal properties to *Da* and where the signal response is highly sensitive to *Da*. This implies that the results of both perfusion images and, as motivated by this study, future simulations in 3D physiologically-realistic geometries will also be sensitive to the estimated value of the Damköhler number. This suggests the need for controlled experiments in order to determine these parameters more precisely. Furthermore, as Damköhler number is a function of velocity, its value will change throughout a heart cycle. Although current imaging protocols that use a temporal resolution of one heart beat generate results that appear to reflect mean flow (Jerosch-Herold et al., 2004), future, novel protocols could conceivably sample multiple times per cycle. In such circumstances, the assumptions underlying the independence of result with cardiac phase might no longer hold and the sensitivity of the signal parameters to *Da* could be significant for the interpretation of those images.

## 7 Diseased case

**Fig. 9:**
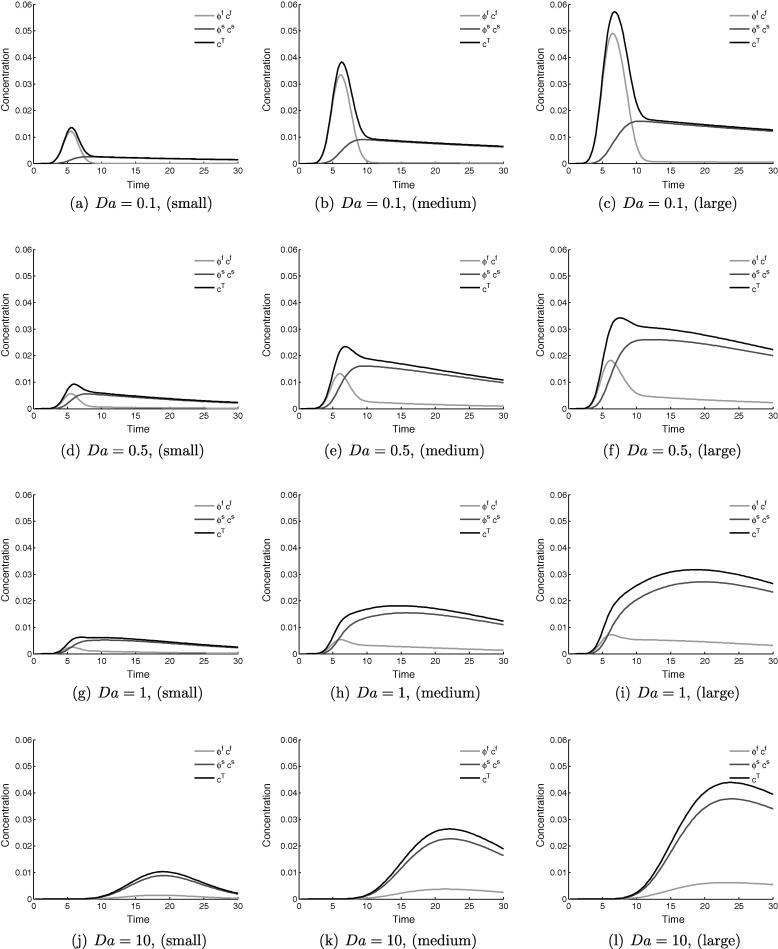
Time series data of the total concentration within the different defects – small (left), medium (middle) and large (right). As the defect size is increased the similarity of the signal trace to the healthy case diminishes, particularly for Da⩾0.5.

Fig. 9 shows time series data for the total concentration in the three different defects, for the following values of Da=0.1,0.5,1 and 10. For Da=0.1, the shape of the curve is much the same as occurs in the point-sampling in the healthy model. This trend is roughly preserved throughout for the smallest defect. This is due to the short diffusion distances allowing the whole area of the defect to behave similarly to healthy tissue, producing false-negative readings.

As the defect size is increased, the spatial inhomogeneity within it increases and for Da⩾0.5 the signals deviate strongly from the point-wise measurements in the healthy case. This is most noticeable in the tail of the curves, which have much slower decay rates than the healthy model.

### 7.1 Effect of varying *Da* and *Dr*

**Fig. 10:**
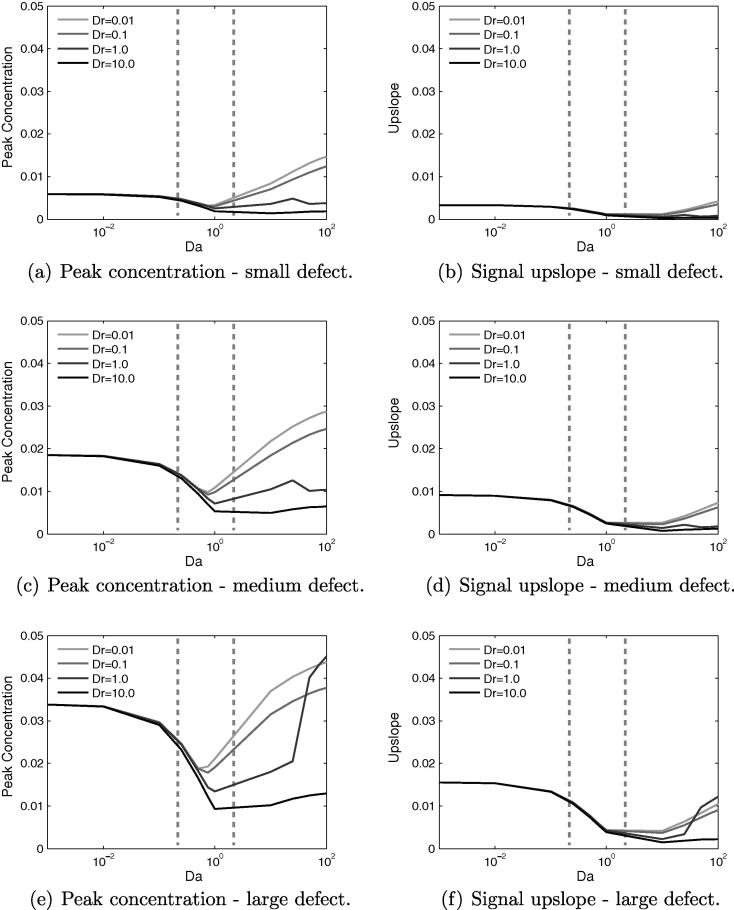
Effects of varying *Da* and *Dr* for three different sizes of perfusion defect, showing that in the diseased model the non-monotonic behaviour in signal properties is centred around a value of Da=1. Estimated current values of *Da* indicated by dashed vertical lines.

Broadly, with a few exceptions, the trends for the peak concentration in the defect case match those for the healthy case. Increasing *Da* initially causes a reduction to a minimum value, followed by a subsequent increase. For the healthy case the minima occurred at Da≈7.5, whereas in the defect case it is mainly Da≈1, as indicated in Fig. 10. This implies that the non-monotonic behaviour will be more significant in the presence of a defect for current contrast agents.

For the upslope value there is negligible increase for Da>1, with the large defect seeing the biggest increase. This is due to the large defect area capturing more of the CA, whereas for the smaller defects it is more readily transported around the defect. This can be understood by considering the limiting case of a defect that spans the width of the domain, in which scenario the bolus has no other path to the outflow but through the defect. Regardless of the behaviour for Da>1, this value marks a distinct change in the signal properties.

Once again, for a given *Da*, increasing *Dr* reduces the peak and upslope due to greater diffusion of the bolus before the defect area. As for the healthy case, this effect is minimal for Da<1, as little contrast agent diffuses into the tissue. For some combinations of *Pe* or *Dr*, and defect size, there is a local maximum in both the peak and upslope at Da≈25. Typically this happens only for Pe⩾1000 or Dr⩽1. The mechanism behind this is difficult to isolate, but is likely due to complex interaction of the various parameters and the defect region, in a manner that cannot occur in the healthy model.

Within the estimated parameter range of *Da* and *Dr*, the variation of both signal quantities is far more dependent on changes in *Da* than on *Dr*, with upslope essentially insensitive to changes in *Dr*. This implies that for current CAs it is the ease with which they permeate through the vessel wall, rather than $D^{s}$, that governs the likelihood of false-negative signals occurring. Furthermore, if using this type of model to aid in detecting perfusion defect size, the significant overlap in the values for peak concentration value could confound analysis, whereas for upslope, the area of overlap is smaller, and confined to values of Da≳0.9 for the medium and large defects only. This again suggests that signal upslope is a more robust indicator of perfusion defect size, than is peak concentration value.

### 7.2 Effect of varying *Da* and *Pe*

**Fig. 11:**
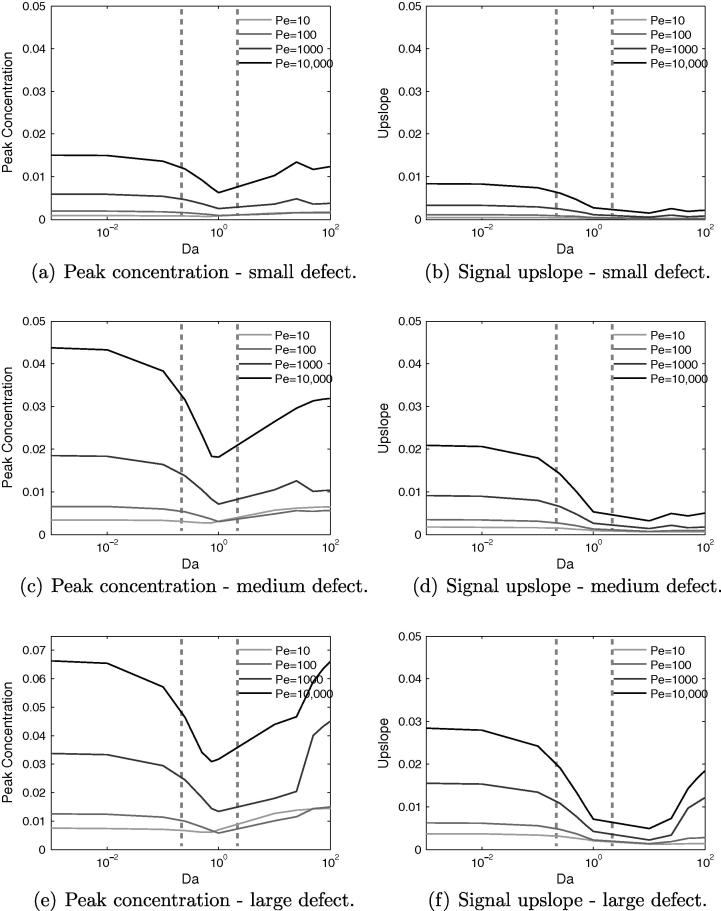
Effects of varying *Da* and *Pe* for three different sizes of perfusion defect, again showing that in the diseased model the non-monotonic behaviour in signal properties is centred around a value of Da=1. Estimated current values of *Da* indicated by dashed vertical lines.

By similar reasoning, for a given value of *Da*, increasing the Peclet number – which can be achieved by a reduction of diffusivity – increases the peak and upslope values. As for the healthy case, this effect occurs for all values of *Da*, as shown in Fig. 11, but only at high values of *Dr*. The trends for varying *Da* and *Pe* remain the same for each defect, but the upslope and peak value increases with defect size, as expected. The main exception is the large defect, for high *Da*, when its spatial dominance ensures that it captures the contrast agent regardless.

Fig. 11 shows that the distinguishability of defect size based on signal characteristics, within the currently relevant range of *Da*, is much improved by increasing the Peclet number, though for signal upslope between Da≈0.8–2, the differences are smaller. Outside this range, for large *Da* this is not so much the case and the discriminatory power of the metric is vastly reduced. These results suggest that there is a benefit to making the diffusion coefficient of the CA in blood as small as possible. However, although a certain amount of extravascular diffusivity is acceptable, the data suggest that Da≈0.8 is a reasonable limit, beyond which confounding effects could start to become large. Finally, the choice of diffusion coefficient within the extracellular space appears to be largely unimportant, particularly for upslope-based quantification methods.

A final point to note is that the Peclet number in question is the global Peclet number based on the fluid velocity and global domain size. However, a local Peclet number can be computed within the defect itself, based on the local velocity, the diffusion coefficient and defect radius. Several computations were performed with different strengths of masking applied to the permeability field in the Darcy flow model, so as to generate flow solutions with different velocities inside the defect area. For a given defect the local Peclet number within the region of the defect showed the expected correlation with the time evolution of the averaged concentration. However, there is no universal trend between the different defects. This can be understood by once again examining Fig. 9, which shows a qualitative change in shape in the time series curves as the defect size is increased.

## 8 Application to imaging practice

In this section the results of the model are applied to address some of the issues and techniques relevant to current clinical imaging.

### 8.1 Nonlinear signal response

**Fig. 12:**
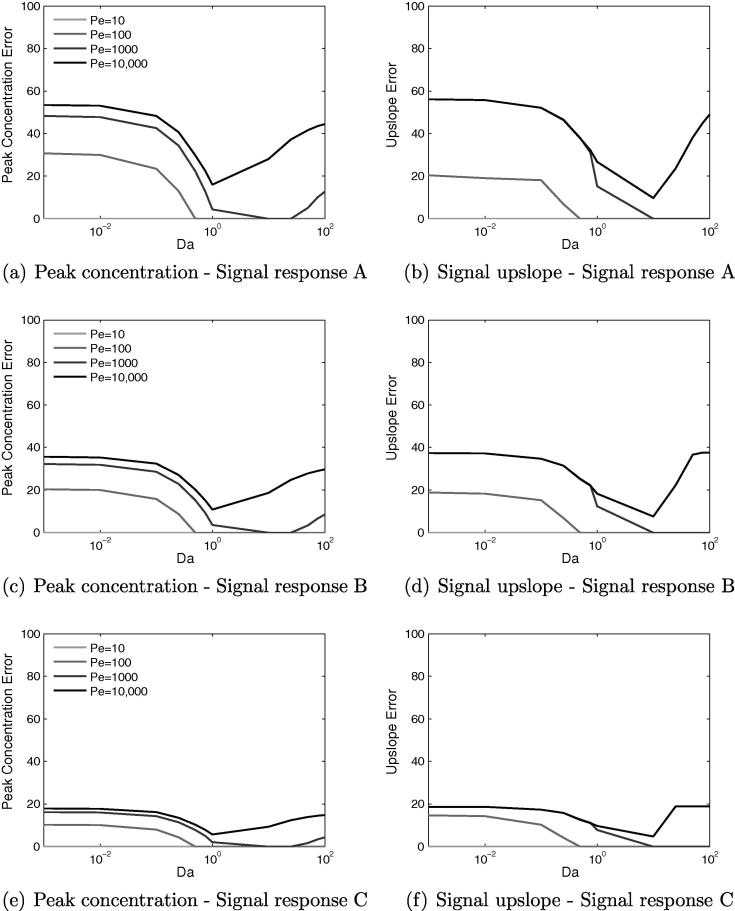
Effects of varying *Da* and *Pe* for three different nonlinear signal response curves. Curve A displays the most nonlinearity and curve C the least. The percentage error between linear and nonlinear signal responses displays non-monotonic behaviour for both peak concentration and upslope. These results suggest that signal nonlinearity is highly significant for contrast agents with very low or high values of *Da*.

Thus far all of the results presented in Sections 6, 7 have been calculated assuming that a linear relationship exists between CA concentration and MR signal. In practice, it is known that these CAs display significant nonlinear behaviour. Therefore, to assess and understand the possible errors that might arise if the assumption of signal linearity is made, three nonlinear signal response curves of varying nonlinearity have been chosen based on the data presented in (Hsu et al., 2008) and used to reprocess the time series data. Peak value and upslope were then recalculated and percentage error between linear and nonlinear values determined, which are shown in Fig. 12.

Signal response A is the most severely nonlinear and signal response C the least nonlinear, and as expected the errors are largest for case A and roughly a factor of 3 smaller for case C. In all cases the error displays the same non-monotonic behaviour as in the underlying trends for peak value and upslope, with the lowest errors occurring for 1<Da<10. For each response curve the percentage errors for peak concentration and upslope are roughly equal for any given pair of *Pe* and *Da*.

With the largest errors occurring for very low or high values of *Da*, the results suggest that particular care and attention is required in practical imaging to correct for the signal nonlinearity, such as with dual-bolus techniques and deconvolution methods. Alternatively, a contrast agent with Da≈1 is much more forgiving from this perspective and in fact for response curve C, the error could be acceptable without any extra correction required. However, given that CAs with low *Da* have other desirable properties it is therefore useful to note that effort spent reducing their nonlinear signal characteristics will be of great benefit to imaging practice.

### 8.2 Fermi deconvolution

The deconvolution approach to perfusion quantification (Hautvast et al., 2012) assumes that the myocardial signal $c_{\mathrm{myo}}$ can be computed by the convolution of an arterial input function $c_{\mathrm{in}}$ and an impulse response function h(t) that represents the myocardial system (Jerosch-Herold et al., 1998).

(15) $$c_{\mathrm{myo}}=\int_{0}^{t}c_{\mathrm{in}}h(\tau -t)\;d\tau =c_{\mathrm{in}}(t)⊗h(t)$$

Various possibilities have been suggested for the choice of h(t), many of which are outlined in (Jerosch-Herold, 2010, Zarinabad et al., 2013). The method studied here assumes that h(t) can be represented by a Fermi function (Jerosch-Herold et al., 1998), a form which has previously been shown to be a reasonable approximation to the myocardial system (Axel, 1983). The Fermi function is defined:

(16) $$h(t)=\frac{F}{\exp(k(t-\tau_{0}-\tau_{\mathrm{onset}}))+1}H(t-\tau_{\mathrm{onset}})$$

where $H(t-\tau_{\mathrm{onset}})$ is the Heaviside step function, *F* is proportional to the flow rate (taken as the peak value of the Fermi function), $\tau_{0}$ sets the width of the function and *k* governs the decay rate. The following minimisation problem:

(17) $$∥c_{\mathrm{myo}}(t)-c_{\mathrm{in}}(t)⊗h(t){∥}^{2}$$

is solved to determine the vales of F,k and $\tau_{0}$, with $\tau_{\mathrm{onset}}$ initially measured from $c_{\mathrm{myo}}$ and $c_{\mathrm{in}}$. The Levenberg–Marquardt nonlinear least squares algorithm was used to minimise Eq. (17).

Traditionally $\tau_{\mathrm{onset}}$ has been solely determined by the user from the point-wise signals, however, Zarinabad et al. (2014) have shown that for point-wise quantification the Fermi fit is very sensitive to the choice of $\tau_{\mathrm{onset}}$, and that by allowing this parameter to vary in an additional outer-loop of minimisation, a better quality of data fitting is possible. Performing the optimisation with $\tau_{\mathrm{onset}}$ fixed produced mixed results, with particularly poor fits for Da<1. Throughout the fitting procedure, it was observed that the results were highly sensitive to the parameter $\tau_{\mathrm{onset}}$, with even small changes from the optimal value causing large deterioration of the fit. However, adopting the method of Zarinabad et al. Zarinabad et al. (2014) greatly improved the quality of fit for all values of *Da*, lending support to the value of this technique.

**Fig. 13:**
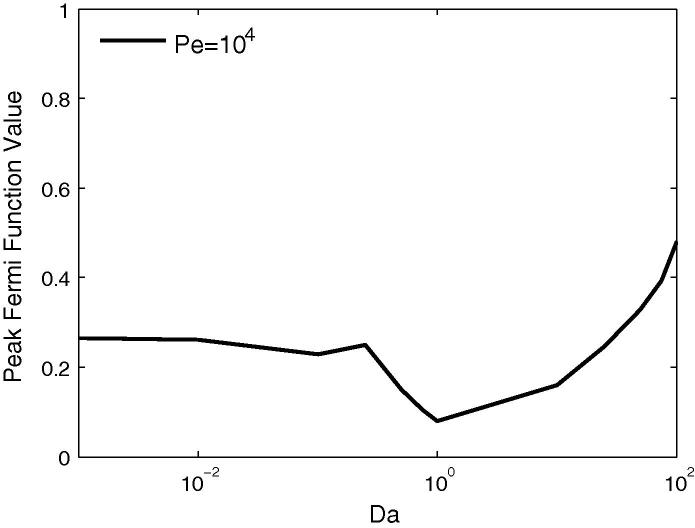
The Fermi deconvolution technique applied to the results from the healthy case, in order to estimate flow rate. The results display the same trend wrt to *Da* as peak value and signal upslope.

The final results of this deconvolution are shown in Fig. 13 which plots the variation of estimated flow rate for varying *Da*, for $\mathrm{Pe}=10^{4}$. The non-monotonic behaviour identified in Fig. 7, Fig. 8 is once again evident, the estimated flow reaching a minimum at Da=1. The fitted function was observed to be very close for Da=100 and still a good fit for Da<0.1 although the upslope of the signal was not accurately represented.

The trough in the curve for 0.1<Da<10 is evidence of the poorest quality curve fits, which is due the Fermi function being unsuitable for matching to a skewed Gaussian function of the kind shown in Fig. 3d and causes an under-estimation of flow in this parameter regime. For the peak magnitude of the Fermi function to accurately represent the peak of the signal, the resultant tail of the predicted signal would greatly overestimate the real tail, causing large errors. The error is observed to be minimised by under-estimating the peak, but fitting much closer to most of the tail. This false-positive effect that occurs with the Fermi function for these values of *Da*, suggests that if contrast agents with these parameters are to be used, then a different deconvolution function should be taken. Alternatively, if Fermi deconvolution is the favoured method, then contrast agents with low values of *Da* should perhaps be used in conjunction with it instead.

### 8.3 Perfusion reserve index

Another clinical metric that is often computed is the perfusion reserve index (Jerosch-Herold et al., 1998), which can be approximated via the normalised ratio of signal upslopes measured during both rest and stress protocols. A detailed examination of this technique is best accomplished using a 3D model in a left ventricle geometry (Nolte et al., 2013) that has been parameterised for both rest and stress physiological conditions. However, the model used here is capable of yielding some preliminary recommendations for regarding the suitability of different contrast agents for use in this technique.

For healthy tissue it is known that during a stress test, the velocity will roughly increase by a factor of three. This implies that *Pe* will likewise triple and that *Da* will be lowered by a third. Therefore when using Fig. 8b to estimate the way upslopes will alter with this change in velocity, both changes with *Pe* and *Da* must be accounted for. For a very low baseline *Pe*, due to a CA with a very large diffusion coefficient, there is little change in upslope with a change in *Pe*, particularly for Da>1 and therefore the perfusion reserve index would potentially be unreliable and susceptible to noise in this parameter range.

However, for Pe>1000, the combination of *Da* and *Pe* means that the biggest changes in upslope, come in the range 0.1<Da<10, i.e. to the left of the minimum point. Conversely, for Da>10, the increase in upslope with *Pe* is partially offset by the reduction in upslope as *Da* reduces. Finally, Da<0.1 sees roughly constant upslope for reducing velocity, and therefore the only change in upslope arises due to the increase in *Pe*. This suggests that a value of *Da* in the range 0.1–10 is likely the best choice for perfusion reserve calculations, in terms of robustness in the presence of noise, followed by values of Da<0.1. Least suitable for this technique are high values of *Da*.

### 8.4 Limitations

The model presented in this study has a number of limitations, foremost among these is the simplified geometry which is unsuitable for simulating patient specific scenarios or for evaluating metrics that inherently rely on physiological geometric detail, such as gradientograms Hautvast et al. (2011). Further, it is possible that in the diseased model the largest defect overestimates the upslope and peak value due to a combination of its size and the imposed-flow boundary condition.

It has been assumed throughout this study that, for both a linear and nonlinear signal response function, the observed MR signal intensity is directly related to the contrast agent concentration. In either case, a phenomenological relationship has been assumed without making attempts to model the various underlying pharmacokinetic processes that generate the resulting MRI signal response Li et al. (2005). This means, for example, that currently the model cannot account for processes such as transcytolemmal water exchange for extra-cellular contrast agents, which has been shown to generate incorrect estimations of signal intensity and other derived quantities Coelho-Filho et al. (2013a).

As used here, the model is currently able to simulate the behaviour of intra-vascular and extra-cellular contrast agents, but not intracellular contrast agents. However, it is worth noting that both the model and our finite element modelling software Cheart are easily able to account for these additional details. For example, to model the transport of intra-cellular contrast agents within the intra-cellular space simply requires the coupling of an additional diffusion equation into the system. Similarly, specific pharmacokinetic effects could also be incorporated into the model via an appropriate system of ODEs, in a similar way to the implementation of the monodomain model of cardiac electrical activation in CHeart Vigueras et al. (2014).

## 9 Conclusions

This study of a simplified model of contrast agent transport in the myocardium has shown the model’s capability for producing physically plausible results in both healthy and disease scenarios. A wide-ranging parameter space study of the four relevant parameters – Peclet number, Damköhler number, diffusivity ratio and fluid porosity – revealed non-monotonic behaviour with respect to the Damköhler number. Furthermore, increased sensitivity of the model to other parameters was observed for large values of *Da*. The high sensitivity of the signal properties to the Damköhler number, particularly around the estimated current value, could present a challenge for formal parameter estimation techniques applied to noisy data. It also suggests that the signal curve properties could change significantly for novel sampling protocols that sample multiple times per heart cycle, which could complicate any future analyses of imaging data that relies on properties such as signal upslope. The results have also shown that, when correlating signal properties to perfusion defect size, the signal upslope is generally a more robust metric than peak concentration value particularly if it were to be used in conjunction with a model-based quantification method under parameter value uncertainty. More specifically, keeping *Da* below 0.8 for any future contrast agents would further improve these properties.

However, the results from Section 8 examining the effect of nonlinear signal response, flow quantification via Fermi deconvolution and the perfusion reserve index, have shown that there is no single best set of contrast agent parameters. The transport properties of the contrast agents interact in different ways with these different aspects of practical imaging. Therefore, these findings should be used to design or select the appropriate contrast agent for a specific imaging protocol and post-processing method. Alternatively for a given contract agent the results reveal which aspects of the imaging process will require the most care in order to achieve the best results.

In future studies these findings will be applied to better understand the behaviour of 3D physiologically-realistic simulations in left-ventricle geometries, but with the knowledge that the results will be sensitive to the choice of contrast agent through-wall diffusivity.

## References

[b0005] Aquaro G.D., Todiere G., Di Bella G., Guiducci L., Pingitore A., Lionetti V. (2013). A fast and effective method of quantifying myocardial perfusion by magnetic resonance imaging. Int. J. Cardiovasc. Imaging.

[b0010] Axel L. (1983). Tissue mean transit time from dynamic computed tomography by a simple deconvolution technique. Invest. Radiol..

[b0015] Barkhausen J., Hunold P., Jochims M., Debatin J.F. (2004). Imaging of myocardial perfusion with magnetic resonance. J. Magn. Reson. Imaging.

[b0020] Beaudoin A., Dreuzy J.-R., Erhel J., Kermarrec A.-M., Bougé L., Priol T. (2007). Euro-Par 2007 Parallel Processing.

[b0025] Beller G.A., Zaret B.L. (2000). Contributions of nuclear cardiology to diagnosis and prognosis of patients with coronary artery disease. Circulation.

[b0030] Brooks A.N., Hughes T.J.R. (1982). Streamline Upwind/Petrov–Galerkin formulations for convection dominated flows with particular emphasis on the incompressible Navier–Stokes equations. Comput. Methods Appl. Mech. Eng..

[b0035] Chapelle, D., Moireau, P., 2014. General coupling of porous flows and hyperelastic formulations – from thermodynamics principles to energy balance and compatible time schemes. Eur. J. Mech. – B/FluidsUpdated version of previously published research report. URL <http://hal.inria.fr/inria-00520612>

[b0040] Chapelle D., Gerbeau J.-F., Sainte-Marie J., Vignon-Clementel I. (2010). A poroelastic model valid in large strains with applications to perfusion in cardiac modeling. Comput. Mech..

[b0045] Chiribiri A., Schuster A., Ishida M., Hautvast G., Nooralipour N.Z., Paul M., Hussain S., Batchelor P., Breeuwer M., Schaeffter T., Nagel E. (2011). Dynamic simulation of first pass myocardial perfusion MR with a novel perfusion phantom. J. Cardiovasc. Magn. Reson..

[b0050] Coelho-Filho O.R., Mongeon F.-P., Mitchell R., Moreno H.J., Nadruz W.J., Kwong R., Jerosch-Herold M. (2013). Role of transcytolemmal water-exchange in magnetic resonance measurements of diffuse myocardial fibrosis in hypertensive heart disease. Circ. Cardiovasc. Imaging.

[b0055] Coelho-Filho O.R., Rickers C., Kwong R.Y., Jerosch-Herold M. (2013). MR myocardial perfusion imaging. Radiology.

[b0060] Cookson A.N., Lee J., Michler C., Chabiniok R., Hyde E., Nordsletten D.A., Sinclair M., Siebes M., Smith N.P. (2012). A novel porous mechanical framework for modelling the interaction between coronary perfusion and myocardial mechanics. J. Biomech..

[b0065] Di Bella E.V.R., Parker D.L., Sinusas A.J. (2005). On the dark rim artifact in dynamic contrast-enhanced MRI myocardial perfusion studies. Magn. Reson. Med..

[b0070] Frangogiannis N.G. (2003). The pathological basis of myocardial hibernation. Histol. Histopathol..

[b0075] Gerke H.H., van Genuchten M.T. (1993). Evaluation of a first-order water transfer term for variably saturated dual-porosity flow models. Water Resour. Res..

[b0080] Grathwohl P. (1998).

[b0085] Hautvast G.L.T.F., Chiribiri A., Lockie T., Breeuwer M., Nagel E., Plein S. (2011). Quantitative analysis of transmural gradients in myocardial perfusion magnetic resonance images. Magn. Reson. Med..

[b0090] Hautvast G., Chiribiri A., Zarinabad N., Schuster A., Breeuwer M., Nagel E. (2012). Myocardial blood flow quantification from MRI by deconvolution using an exponential approximation basis. IEEE Trans. Biomed. Eng..

[b0095] Hsu L.-Y., Kellman P., Arai A.E. (2008). Nonlinear myocardial signal intensity correction improves quantification of contrast-enhanced first-pass MR perfusion in humans. J. Magn. Reson. Imaging.

[b0100] Huyghe J.M., Arts T., van Campen D.H., Reneman R.S. (1992). Porous medium finite element model of the beating left ventricle. Am. J. Physiol. – Heart Circul. Physiol..

[b0105] Hyde E.R., Cookson A.N., Lee J., Michler C., Goyal A., Sochi T., Chabiniok R., Sinclair M., Nordsletten D.A., Spaan J., van den Wijngaard J.P., Siebes M., Smith N.P. (2013). Multi-scale parameterisation of a myocardial perfusion model using whole-organ arterial networks. Ann. Biomed. Eng..

[b0110] Hyde E.R., Michler C., Lee J., Cookson A.N., Chabiniok R., Nordsletten D.A., Smith N.P. (2013). Parameterisation of multi-scale continuum perfusion models from discrete vascular networks. Med. Biol. Eng. Comput..

[b0115] Ishida M., Sakuma H., Murashima S., Nishida J., Senga M., Kobayasi S., Takeda K., Kato N. (2009). Absolute blood contrast concentration and blood signal saturation on myocardial perfusion MRI: estimation from CT data. J. Magn. Reson. Imaging.

[b0120] Ishida M., Schuster A., Morton G., Chiribiri A., Hussain S., Paul M., Merkle N., Steen H., Lossnitzer D., Schnackenburg B., Alfakih K., Plein S., Nagel E. (2011). Development of a universal dual-bolus injection scheme for the quantitative assessment of myocardial perfusion cardiovascular magnetic resonance. J. Cardiovasc. Magn. Reson..

[b0125] Jerosch-Herold M. (2010). Quantification of myocardial perfusion by cardiovascular magnetic resonance. J. Cardiovasc. Magn. Reson..

[b0130] Jerosch-Herold M., Wilke N., Stillman A.E. (1998). Magnetic resonance quantification of the myocardial perfusion reserve with a Fermi function model for constrained deconvolution. Med. Phys..

[b0135] Jerosch-Herold M., Seethamraju R.T., Swingen C.M., Wilke N.M., Stillman A.E. (2004). Analysis of myocardial perfusion MRI. J. Magn. Reson. Imaging.

[b0140] Kelle S., Graf K., Dreysse S., Schnackenburg B., Fleck E., Klein C. (2010). Evaluation of contrast wash-in and peak enhancement in adenosine first pass perfusion CMR in patients post bypass surgery. J. Cardiovasc. Magn. Reson..

[b0145] Knesewitsch T., Meierhofer C., Rieger H., Rossler J., Frank M., Martinoff S., Hess J., Stern H., Fratz S. (2013). Demonstration of value of optimizing ECG triggering for cardiovascular magnetic resonance in patients with congenital heart disease. J. Cardiovasc. Magn. Reson..

[b0150] Köstler H., Ritter C., Lipp M., Beer M., Hahn D., Sandstede J. (2008). Comparison of different contrast agents and doses for quantitative MR myocardial perfusion imaging. J. Magn. Reson. Imaging.

[b0155] Li X., Rooney W.D., Springer C.S.J. (2005). A unified magnetic resonance imaging pharmacokinetic theory: intravascular and extracellular contrast reagents. Magn. Reson. Med..

[b0160] Makowski M., Jansen C., Webb I., Chiribiri A., Nagel E., Botnar R., Kozerke S., Plein S. (2010). First-pass contrast-enhanced myocardial perfusion MRI in mice on a 3-T clinical MR scanner. Magn. Reson. Med..

[b0165] May-Newman K., McCulloch A.D. (1998). Homogenization modeling for the mechanics of perfused myocardium. Progr. Biophys. Mol. Biol..

[b0170] McCormick M., Nordsletten D.A., Kay D., Smith N.P. (2013). Simulating left ventricular fluid–solid mechanics through the cardiac cycle under LVAD support. J. Comput. Phys..

[b0175] Michler C., Cookson A.N., Chabiniok R., Hyde E., Lee J., Sinclair M., Sochi T., Goyal A., Vigueras G., Nordsletten D.A., Smith N.P. (2012). A computationally efficient framework for the simulation of cardiac perfusion using a multi-compartment Darcy porous-media flow model. Int. J. Numer. Methods Biomed. Eng..

[b0180] Nolte F., Hyde E.R., Rolandi C., Lee J., van Horssen P., Asrress K., van den Wijngaard J.P.H.M., Cookson A.N., van de Hoef T., Chabiniok R., Razavi R., Michler C., Hautvast G.L.T.F., Piek J.J., Breeuwer M., Siebes M., Nagel E., Smith N.P., Spaan J.A.E. (2013). Myocardial perfusion distribution and coronary arterial pressure and flow signals: clinical relevance in relation to multiscale modeling, a review. Med. Biol. Eng. Comput..

[b0185] Nordsletten D., Kay D., Smith N. (2010). A non-conforming monolithic finite element method for problems of coupled mechanics. J. Comput. Phys..

[b0190] Plein S., Ryf S., Schwitter J., Radjenovic A., Boesiger P., Kozerke S. (2007). Dynamic contrast-enhanced myocardial perfusion MRI accelerated with k-t SENSE. Magn. Reson. Med..

[b0195] Ritter C., Brackertz A., Sandstede J., Beer M., Hahn D., Köstler H. (2006). Absolute quantification of myocardial perfusion under adenosine stress. Magn. Reson. Med..

[b0200] Shipley R., Chapman S. (2010). Multiscale modelling of fluid and drug transport in vascular tumours. Bullet. Math. Biol..

[b0205] Stegmann M., Ólafsdóttir H., Larsson H. (2005). Unsupervised motion-compensation of multi-slice cardiac perfusion MRI. Med. Image Anal..

[b0210] Stinstra J., Roberts S., Pormann J., Macleod R., Henriquez C. (2006). A model of 3D propagation in discrete cardiac tissue. Comput. Cardiol..

[b0215] Su M.-Y.M., Yang K.-C., Wu C.-C., Wu Y.-W., Yu H.-Y., Tseng R.-Y., Tseng W.-Y.I. (2007). First-pass myocardial perfusion cardiovascular magnetic resonance at 3 Tesla. J. Cardiovasc. Magn. Reson..

[b0220] Tofts P.S., Brix G., Buckley D.L., Evelhoch J.L., Henderson E., Knopp M.V., Larsson H.B., Lee T.-Y., Mayr N.A., Parker G.J., Port R.E., Taylor J., Weisskoff R.M. (1999). Estimating kinetic parameters from dynamic contrast-enhanced T1-weighted MRI of a diffusable tracer: standardized quantities and symbols. J. Magn. Reson. Imaging.

[b0225] Townsend, N., Wickramasinghe, K., Bhatnagar, P., Smolina, K., Nichols, M., Leal, J., Luengo-Fernandez, R., Rayner, M., 2012. Coronary Heart Disease Statistics 2012 Edition. British Heart Foundation, London.

[b0230] van den Wijngaard J.P., Schwarz J.C., van Horssen P., van Lier M.G., Dobbe J.G., Spaan J.A., Siebes M. (2013). 3D imaging of vascular networks for biophysical modeling of perfusion distribution within the heart. J. Biomech..

[b0235] Vankan W.J., Huyghe J.M., Drost M.R., Janssen J.D., Huson A. (1997). A finite element mixture model for hierarchical porous media. Int. J. Numer. Methods Eng..

[b0240] Vigueras G., Roy I., Cookson A., Lee J., Smith N., Nordsletten D. (2014). Toward gpgpu accelerated human electromechanical cardiac simulations. Int. J. Numer. Biomed. Eng..

[b0245] Zarinabad N., Chiribiri A., Hautvast G.L.T.F., Ishida M., Schuster A., Cvetkovic Z., Batchelor P.G., Nagel E. (2012). Voxel-wise quantification of myocardial perfusion by cardiac magnetic resonance. Feasibility and methods comparison. Magn. Reson. Med..

[b0250] Zarinabad N., Chiribiri A., Hautvast G., Shuster A., Sinclair M., Wijngaard J., Smith N., Spaan J., Siebes M., Breeuwer M., Nagel E., Ourselin S., Rueckert D., Smith N. (2013). Functional Imaging and Modeling of the Heart.

[b0255] Zarinabad, N., Hautvast, G.L.T.F., Sammut, E., Arujuna, A., Breeuwer, M., Nagel, E., Chiribiri, A., 2014. Effects of tracer arrival time on the accuracy of high-resolution (voxel-wise) myocardial perfusion maps from contrast-enhanced first-pass perfusion. Under Rev. IEEE TBME. 10.1109/TBME.2014.2322937 PMC7611159 24833413

